# Cross-sectional analysis of association between socioeconomic status and utilization of primary total hip joint replacements 2006–7: Australian Orthopaedic Association National Joint Replacement Registry

**DOI:** 10.1186/1471-2474-13-63

**Published:** 2012-04-30

**Authors:** Sharon L Brennan, Tyman Stanford, Anita E Wluka, Margaret J Henry, Richard S Page, Stephen E Graves, Mark A Kotowicz, Geoffrey C Nicholson, Julie A Pasco

**Affiliations:** 1 Barwon Epidemiology and Biostatistics Unit, Barwon Health, Deakin University, Kitchener House, PO Box 281, Geelong, Victoria, 3220, Australia; 2 North West Academic Centre, Department of Medicine, The University of Melbourne Western Health, 176 Furlong Rd, St Albans, VIC, 3021, Australia; 3 Data Management and Analysis Centre, Discipline of Public Health, University of Adelaide, MDP DX650, Adelaide, SA, 5005, Australia; 4 Department of Epidemiology and Preventive Medicine, Monash University, Alfred Centre, 89 Commercial Road, Melbourne, VIC, 3004, Australia; 5 Barwon Orthopaedic Research Unit, Barwon Health, Ryrie Street, Geelong, VIC, 3220, Australia; 6 Australian Orthopaedic Association Joint Replacement Registry, MDP DX650, Adelaide, SA, 5005, Australia; 7 Department of Endocrinology and Diabetes, Barwon Health, Ryrie Street, Geelong, VIC, 3220, Australia; 8 Rural Clinical School, The University of Queensland, Locked Bag 9009, Toowoomba, DC QLD, 4350, Australia

**Keywords:** Hip joint replacement, Socioeconomic status, Utilization, Australia

## Abstract

**Background:**

The utilization of total hip replacement (THR) surgery is rapidly increasing, however few data examine whether these procedures are associated with socioeconomic status (SES) within Australia. This study examined primary THR across SES for both genders for the Barwon Statistical Division (BSD) of Victoria, Australia.

**Methods:**

Using the Australian Orthopaedic Association National Joint Replacement Registry data for 2006–7, primary THR with a diagnosis of osteoarthritis (OA) among residents of the BSD was ascertained. The Index of Relative Socioeconomic Disadvantage was used to measure SES; determined by matching residential addresses with Australian Bureau of Statistics census data. The data were categorised into quintiles; quintile 1 indicating the most disadvantaged. Age- and sex-specific rates of primary THR per 1,000 person years were reported for 10-year age bands using the total population at risk.

**Results:**

Females accounted for 46.9% of the 642 primary THR performed during 2006–7. THR utilization per 1,000 person years was 1.9 for males and 1.5 for females. The highest utilization of primary THR was observed in those aged 70–79 years (males 6.1, and females 5.4 per 1,000 person years). Overall, the U-shaped pattern of THR across SES gave the appearance of bimodality for both males and females, whereby rates were greater for both the most disadvantaged and least disadvantaged groups.

**Conclusions:**

Further work on a larger scale is required to determine whether relationships between SES and THR utilization for the diagnosis of OA is attributable to lifestyle factors related to SES, or alternatively reflects geographic and health system biases. Identifying contributing factors associated with SES may enhance resource planning and enable more effective and focussed preventive strategies for hip OA.

## Background

Total hip replacement (THR) is a cost-effective elective procedure undertaken to relieve pain and improve quality of life for severe end-stage arthritis [1,2], with the rate for primary THR in Australia for 2005–2006 reported as 102.2 per 100,000 [3]. Variations in the utilization of THR procedures have been reported across socioeconomic status (SES) in various countries such as England [4-8], USA [9], Italy [10], and other geographic regions [11-15], although the latter has often been explained in relation to urban and rural distinctions, and as such suggested to be associated with access issues. Yet a large study from the USA has recently shown disparities in THR surgery across age, gender, and income levels [4,16], suggesting that rather than geographic location *per se,* it may be that social demographics influence THR utilization. Furthermore, some USA studies have identified racial and ethnic disparities in the access and use of THR [12,17,18], potentially driven by psychosocial factors and beliefs, and which may be plausibly related to greater social disadvantage in some groups. Indeed, ethnicity is often conflated with SES [19].

In Australia, compared to other countries, there are few data examining SES and joint replacement. The well funded, universal, accessible Australian public health system in conjunction with private health system options should, in theory, result in few disparities in utilization of health care across the country. Yet a study that examined hospital separations for primary joint replacement surgeries identified a lower rate of THR for individuals residing in more socially disadvantaged areas of Australia [15]. Access to health care and THR utilization across different SES groups occurs due to different social and economic imperatives in different social groups. For example, the less disadvantaged individual may undergo THR when personal schedules allow, whereas the more disadvantaged individual relies upon vacancies in the public surgery waiting list. Thus, understanding the association between SES and THR utilization has an important implication for the promotion and allocation of health services resources across the social spectrum.

Linked with SES are occupation types [20-22], and these, along with related lifestyle, demographic and body composition data have been well described for the Barwon Statistical Division (BSD) and shown to be representative of the broader Australian population. The BSD has one major city, Geelong, and is located in the State of Victoria, Australia. Combined with the immediate suburbs, Geelong constitutes the third largest non-capital city in Australia. Also included within the BSD are coastal resort towns, small acreage properties, larger more traditional farms, and small townships based upon agricultural and tourist industries. Statistical Divisions are large, general purpose, regional type geographic areas defined by the Australian Bureau of Statistics (ABS), and the application of Statistical Divisions to examine area-based SES provides a stable, base spatial unit to examine associations between health and social disadvantage within each State or Territory of Australia [23]. Using ABS Census data, a comparison between BSD and Australian demographics has shown that differences in females did not exceed 1.7% for age profiles, 6.5% for country of birth, 3.2% for marital status, 3.0% for types of employment, and 2.5% for weekly income [24]. Similarly, males residing in the BSD region are representative of the broader Australian population, especially with respect to the majority population of mixed European ancestry [25]. The BSD is an excellent region for epidemiological research as it encompasses areas across the full spectrum of SES, and comprises both urban and rural regions.

To the best of our knowledge there are few data examining whether utilization of THR procedures are associated with area-based SES in Australia using a comprehensive data registry, and none specifically focused upon the BSD; a region in which a large body of work exists with regards to other musculoskeletal disorders [26,27], and which has been shown as representative of the broader Australian population [24,28]. Using a comprehensive registry of hip replacement surgeries across Australia, we focused on a representative region of broader Australia to assess the association between SES and utilization of THR in both males and females for the BSD.

## Methods

### Total hip joint replacement

Incident hip joint replacements for 2006–7 were identified from the Australian Orthopaedic Association National Joint Replacement Registry (AOANJRR). The registry commenced in September 1999, funded by the Commonwealth Government through the Department of Health and Ageing, and was introduced in a state-by-state approach that was completed nationally in 2002. Collection of Victorian data commenced in 2001. The AOANJRR monitors the performance and outcome of hip and knee replacement surgery Australia-wide and receives voluntary cooperation from all hospitals undertaking joint replacement surgeries performed within both the public and private health systems. The database has been validated against health department unit record data using a sequential multi-level matching process and, coupled with the retrieval of unreported procedures, the AOANJRR is the most complete set of data relating to hip and knee replacement in Australia.

Primary THR was defined as primary replacement of the acetabulum and femoral articular surface. Both conventional and resurfacing procedures were included. Primary partial hip replacements performed during 2006–7 were excluded.

All subjects who underwent a primary THR for a diagnosis of osteoarthritis (OA) during 2006–7 and whose residential postcode was identified as within the BSD of Victoria were eligible for inclusion. Of the 874 primary THR procedures fulfilling these criteria, 73.5% (n = 642) had a diagnosis of OA. The remaining primary THR were for neck of femur fracture (21.2%), avascular necrosis (1.4%), or other reasons including developmental dysplasia, tumour, rheumatoid arthritis, other inflammatory arthritis, failed internal fixation, or arthrodesis takedown (2.6%).

### Socioeconomic status

Once joint replacements were identified by postcode from the AOANJRR, the full residential address of each subject was matched to the corresponding ABS Census Collection District, an area of approximately 250 households. ABS reference data were used to determine the Socio-Economic Indexes For Areas (SEIFA) value from the 2006 census for each subject. The Index of Relative Socioeconomic Disadvantage (IRSD) was applied for this analysis, in which quintile 1 represented the most disadvantaged and quintile 5 represented the least disadvantaged. Validation of the SEIFA index was undertaken by analysts from the ABS Regional Offices, and also an external peer review of the variables and methodology used in SEIFA 2006 was performed by a group of academic and policy research experts who were skilled in socioeconomic modelling and analysis [29]. Variables included in the SEIFA (numerators and denominators) were validated by summing SEIFA variables at the small area to the State totals, which were then compared to published or independently created figures [29]. The ABS indicates principal components analysis, the technique applied to develop and weight the scores, has shown to be reliable [29,30]. SEIFA values were unavailable for 59 subjects (9.0% representing 0.03% of the total population at risk) and were thus excluded from final analysis. In 2006, approximately 3% of Census Collection Districts could not be given a SEIFA score for reasons which included: fewer than ten people residing in an area, fewer than five employed people in an area, five or fewer occupied private dwellings in an area, or areas in which non-response to Census questions including occupation, labour force status, type of educational institution attending, or non-school qualifications exceeded 70% [31]. The AOANJRR Data Review Committee approved the study.

### Statistical analysis

Primary THR rates were calculated for 10-year age strata for men and women and expressed as the number of procedures per 1,000 persons per year, based on 2006 ABS Census population figures for each SES quintile.

We used Poisson regression to model the relative risk of primary THR per unit time stratified by gender across SES quintiles, and adjusted for age (as a categorical variable). Given the interaction between SES and gender, the model was set up as:

log(N) = log(PAR) + intercept + age group + SES + gender + SES x gender + error

In order to examine whether the rates of THR varied within gender across different SES quintiles, post-hoc comparisons were created of estimated N/PAR within gender across different SES quintiles (alpha = 0.05). Goodness of fit and assumptions of the model were tested using the Residual Quantile-Quantile Plot to examine normality; the plot was shown to be linear. The Residual versus Predicted Plot was used to check the constant variance of residuals; a random scatter was observed with no systematic trends, although a slight fanning of the residuals could be considered. Analyses were performed using SAS version 9.2 (SAS Institute Inc., Cary, North Carolina).

## Results

Of the 642 primary THR with a diagnosis of OA, females accounted for 46.9%. Table 1 presents the crude data and age-stratified rates of THR utilization per 1,000 person years for each gender and SES quintile. THR utilization per 1,000 person years was 1.9 for males, and 1.5 for females. The highest utilization of primary THR was observed in those aged 70–79 years (males 6.1, and females 5.4 per 1,000 person years).

**Table 1:** CRUDE numbers and rates per 1,000py of total hip replacements (thr) by age, gender and socioeconomic quintiles for 2006–7

		**Total**			**Quintile 1**			**Quintile 2**			**Quintile 3**			**Quintile 4**			**Quintile 5**	
Age (yrs)	At risk	n= THR^a^	Rate	At risk	n	Rate	At risk	n	Rate	At risk	n	Rate	At risk	n	Rate	At risk	n	Rate
**Males**																		
20-29	14,727	-	-	3167	-	-	2998	-	-	3071	-	-	3120	-	-	2371	-	-
30-39	16,624	3	0.1	3173	-	-	3149	-	-	3638	-	-	3746	3	0.4	2918	-	-
40-49	18,192	29	0.8	3315	2	0.3	3155	5	0.8	3922	3	0.4	4011	6	0.7	3789	7	0.9
50-59	17,020	56	1.6	2992	37	6.2	3012	6	1.0	3758	13	1.7	3572	11	1.5	2686	17	2.3
60-69	11,767	115	4.9	2262	20	4.4	2354	28	5.9	295	14	2.9	2410	27	5.6	2346	17	3.6
70-79	8,383	102	6.1	1855	20	5.4	1864	24	6.4	1682	14	4.2	1644	19	5.8	1338	17	6.4
>79	4,361	36	4.1	876	4	2.3	1009	8	4.0	1004	9	4.5	816	4	2.5	656	8	6.1
**Females**																		
20-29	14,498	-	-	3300	-	-	2975	-	-	2984	-	-	2914	-	-	2325	-	-
30-39	17,599	2	0.06	3417	-	-	3274	2	0.3	3817	-	-	3834	-	-	3257	-	-
40-49	19,367	10	0.3	3509	3	0.4	3388	-	-	4169	1	0.1	4140	2	0.2	4161	3	0.4
50-59	17,653	54	1.5	3242	13	2.0	3235	9	1.4	3836	5	0.7	3666	11	1.5	3674	10	1.4
60-69	12,435	85	3.4	2552	14	2.7	2506	9	1.8	2596	19	3.7	2499	14	2.8	2282	24	5.3
70-79	9,872	107	5.4	2230	20	4.5	2248	25	5.6	2104	19	4.5	1808	19	5.3	1482	18	6.1
>79	7,320	43	2.9	1480	9	3.0	1711	10	2.9	1698	7	2.1	1399	9	3.2	1032	8	3.9

^a^ n = Total hip replacement (THR) in the Total Column includes Primary THR where SEIFA values were unavailable.

*Most disadvantaged quintile of SES. Rate = THR per 1,000 person years. At risk = population at risk.

**Figure 1:**
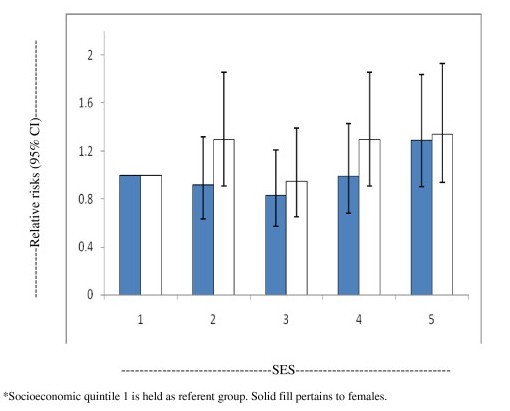
**Age-adjusted relative risks (95%ci) of total hip joint replacement stratified by gender and across socioeconomic quintiles for 2006-7.** *Socioeconomic quintile 1 is held as referent group. Solid fill pertains to females.

Figure 1 presents the gender-specific, age-adjusted relative risks of THR utilization. For females, there was a non-significant estimated increased rate in primary THR for SES quintile 5 when compared to SES quintile 1. Females in quintile 1 had an estimated increased rate of THR compared to all other SES quintiles; however these patterns of increased rates were not significant. For males, all SES quintiles with the exception of quintile 3 had an estimated increased rate of THR compared to quintile 1; however, these were also non-significant. No significant results at the alpha = 0.05 level were observed from the post-hoc comparisons. Overall, the U-shaped pattern of THR across SES gave the appearance of bimodality for both males and females, whereby rates were greater for both the most disadvantaged and least disadvantaged groups; the U-shaped pattern of association was more marked for females.

## Discussion

The results of this study suggest non-significant U-shaped patterns of association exist for utilization of THR performed for osteoarthritis across SES quintiles for males, and also for females, which appeared at greater rates in both upper and lower SES quintiles. The highest rates of THR utilization for both genders were observed in the age group of 70–79 years.

Whereas females have, in general, a higher prevalence of OA compared to males [32,33], it has been suggested that a greater proportion of THR procedures for OA occur in males rather than females [14]; these data suggest that females are under treated [4]. Indeed, it has been reported females are less likely to; consult their general practitioner for hip OA, be referred for specialist care, consult an orthopaedic surgeon, be on a waiting list for primary THR, or females may be more hesitant to undergo primary THR than males [34]. A recent review has demonstrated significant socioeconomic and geographic variation in the frequency of THR [35]. The U-shape pattern of association between THR and SES observed for both sexes within this study may be explained by considering that a differential referral pattern for THR may indeed exist between SES [35], related to public versus private health care usage [36].

Lifestyle behaviours and body composition of the adult population of BSD have previously been reported using large representative cohorts of males and females [37,38], and these data provide some insight into potential explanations for our observations. A clear inverse gradient between obesity and SES has been previously reported in the BSD for both genders [37,38], and the association between obesity and cartilage loss associated with osteoarthritis is well-documented [39,40], as is the association between obesity and joint replacement for end-stage osteoarthritis [41]. It is notable in the male population of the BSD that a higher proportion of tradespersons are in quintiles 2 and 4 [37], replicating the peaks of THR in males observed in this study. Furthermore, given that males in quintile 1 were more likely to undergo THR at a younger age (50–59 years) compared to males in other quintiles, this may reflect both the reported association between strenuous occupations and THR for hip OA [42], and the greater number of manual labourers and related workers in quintile 1 within the BSD compared to any other quintile [37]. Among females greater levels of vigorous activity are undertaken by those in quintile 5 [38]. Thus we may speculate that a greater risk of hip OA may be related to the type and/or level of activity undertaken, whether leisure or occupation related.

Our study addressed the question of THR utilization, rather than access to the health system. However, it is important to recognise that differences in health-seeking behaviour may exist between SES quintiles, whereby those in greatest need exist in the lower SES, whereas those at various levels of need are represented in the upper SES groups. Related to this point is the severity of hip OA, and the willingness of the patient to consider joint replacement [43], especially for those of lower SES [34], and expectations by both medical professionals and the patients themselves of poorer outcomes [43]. This latter point may be more associated with lower SES, and related to concerns of co-morbidities [44]. Furthermore, waiting times for THR through the public system may influence the utilization of THR for individuals of lower SES, and also for individuals in other SES quintiles with low or no private health coverage. Thus, utilization of THR is determined partly by access, and partly by choice, and indeed between countries given that differences in healthcare systems will also exist from one country to the next. Gender differences may be inherent in these issues. Whilst these explanations may begin to account for the lack of a significant association between quintiles of SES and utilization of THR, these data may partly explain the U-shape pattern of association we observed and also support the possibility that differences in accessibility issues to THR exist across SES groups. For instance, whereas hip OA has been associated with lower SES [45], THR rates in our study appeared higher at both ends of the SES continuum, not only for the lower SES groups.

A strength of this study was that the primary THR were ascertained from a comprehensive national registry that has been validated against health department unit record data using a sequential multi-level matching process. Coupled with the retrieval of unreported procedures, the registry is the most complete set of data relating to hip replacement in Australia. The BSD is a region that is ideal for epidemiological studies, having a single major public hospital and a relatively stable population as previously reported [46]. The examination of SES within a defined Statistical Division ensures the social and economic links, which characterise the study population, are unified under the influence of one major town [23]. Of the primary THR cases identified for 2006–7 in the BSD, 9% could not be coded for SES, which may have influenced our findings, for instance it may be possible that the excluded Census Collection Districts with <5 employed people were the most disadvantaged areas. However, given that the spread of these patients was relatively equal between genders (57% male), and represented equal proportions of the population for each gender (both 0.03%), any potential disparity would be non-differential. The use of public *versus* private health system has previously been associated with THR [36], with a greater proportion of Australian THR performed in the private system [47], yet is a potential unmeasured confounder in our analyses. However, the goal of this study was to examine utilization of THR across SES rather than accessibility to THR which may be increased for those that have private health coverage compared to those that rely upon the public health care system. The IRSD is an aggregate of various individual parameters of SES and is formed into an area-based measure of SES from data collected as part of the Australian Census. We acknowledge that the use of an aggregate SES index assumes that relatively disadvantaged individuals do not reside in areas of upper SES. This study is an analysis of data from one region, and thus these findings cannot be assumed to exist in other geographic regions of Australia, or relate to the country as a whole. Our classification of Caucasian may have limited the ability to examine any ethnic or cultural differences in THR, as has been shown in previous studies [18,36,48]. Numbers may have limited our analysis, especially given that THR utilization was stratified by quintiles of SES and gender.

## Conclusion

It is imperative that this analysis of AOANJRR data be performed on a larger scale, to elucidate the association between SES, gender and utilization of THR for the broader Australian population.

## Competing interests

SLB, TS, MJH, MAK, GCN, and JAP have no competing interests. AEW is a Committee member of the Scientific Programme and Research Committee, Australian Rheumatology Association, and is on the Advisory Board of the Australian Rheumatology Association Research Trust Scientific Advisory Board. RSP has received research support from Ascension Orthopaedics, and financial support from Synthes, and De Puy Education for Fellowship Position. RSP is a Committee member of the Australian Orthopaedic Association National Joint Replacement Registry (AOANJRR), from which the dataset used for this analysis was extracted, and is an Executive of the Shoulder and Elbow Society. SEG is the Director of the AOANJRR, from which the dataset used for this analysis was extracted.

## Authors’ contributions

All authors participated in the design of the study. SLB drafted the manuscript. TS performed the statistical analysis, and SEG and SLB supervised the analysis. SLB, MH, MAK, GCN, AEW, RSP, and JAP guided and reviewed the manuscript. All authors read and approved the final manuscript.

## Pre-publication history

The pre-publication history for this paper can be accessed here:

http://www.biomedcentral.com/1471-2474/13/63/prepub
